# A novel blended and interprofessional approach to pediatric emergency training: self-assessment, perception, and perceived long-term effects

**DOI:** 10.1186/s12909-024-06381-3

**Published:** 2024-11-28

**Authors:** Ronny Lehmann, Michaela Klinke Petrowsky, Anke Seitz, Jochen Meyburg, Walter Eppich, Georg F. Hoffmann, Burkhard Tönshoff, Sören Huwendiek

**Affiliations:** 1 Department of Pediatrics I, Center for Pediatrics and Adolescent Medicine, University Hospital Heidelberg, Im Neuenheimer Feld 430, 69120 Heidelberg, Germany; 2 UMM Klinik für Kinderchirurgie, Theodor-Kutzer-Ufer 1-3, 68167 Mannheim, Germany; 3 Kinder- und Jugendarztpraxis Dr. Seitz, Werderstraße 3, 88348 Bad Saulgau, Germany; 4 Klinik für Kinder- und Jugendmedizin, Klinikum Ludwigsburg, Posilipostr. 4, 71640 Ludwigsburg, Germany; 5 RCSI SIM Centre for Simulation Education and Research, RCSI University of Medicine and Health Sciences, Dublin, Ireland; 6 Institute for Medical Education, Department for Assessment and Evaluation, Mittelstrasse 43, Bern, 3012 Switzerland

**Keywords:** Pediatric emergency care, Interprofessional education, Blended learning, Virtual patients, Team training, Simulation

## Abstract

**Background:**

We developed a blended training program at a tertiary pediatric center based on hospital-specific emergency guidelines, profession-specific Virtual Patients (VPs), and interprofessional team training. Using this novel approach, we addressed differing educational needs of medical and nursing staff and intrinsic cognitive overload among participants, aiming for harmonization of in-house emergency proceedings.

**Methods:**

Self-assessments of pediatric emergency knowledge and skills were conducted before (T1) and after (T2) preparation using VPs, as well as after the team training day (T3). At T3, participants completed questionnaires on the training approach, its components, and learning impact. Ten months after the training, a follow-up survey (T4) queried perceived benefits within and beyond emergency situations.

**Results:**

A total of 56 medical staff and 56 nursing staff members participated in the pilot phase. Of these, *N* = 55 (98%) and *N* = 48 (85%), respectively, returned self-assessments; questionnaires were completed by *N* = 55 (98%) and *N* = 51 (91%), respectively. In both groups, 57 participants (50.9%) completed the follow-up survey. After team training (T3), both groups had statistically significant increased knowledge and skill scores compared with those at T1. Regarding the blended approach and its components, medical and nursing staff alike rated the entire course and its guidelines, the preparatory VPs, and the team training very highly. Participants felt being better prepared for pediatric emergencies. Perceived strengths of the training approach were in the triangulation of teaching methods and its interprofessionalism. More training scenarios were requested, as well as recurrent training. In the follow-up, participants reported improved confidence and calmness, as well as improved communication and collaboration when involved in an emergency. Beyond emergencies, benefits were reported in daily routines.

**Conclusions:**

Our blended approach was perceived as being effective in improving preparedness among medical and nursing house staff. This approach permits customization of content and deliberate practice to improve pediatric critical care.

**Supplementary Information:**

The online version contains supplementary material available at 10.1186/s12909-024-06381-3.

## Background

To improve emergency preparedness among professional staff, simulation-based team training has gained momentum in health professions curricula and continuing professional development [1–4]. Instructional design is a key topic in simulation-based research for improving such educational approaches [5]. Whereas there is consensus regarding team training being crucial for reducing errors and ensuring patient safety [6–8], instructional designs must be optimized to improve team behaviors [9].

Regarding instructional design approaches to emergency team trainings, teams face three challenges: (a) differing educational needs amongst the professions involved (often medical and nursing staff), (b) intrinsic cognitive overload among training participants, and at times, (c) a lack of uniform treatment guidelines even within a single institution. We expand on these challenges below.

First, educational needs vary across health care professions. Interprofessional education is increasingly focused on improving understanding of its own role in patient care and the role of other professionals to improve team collaboration and communication [6, 10]. Physicians are often the focus of medical education research; few studies have evaluated training programs for nursing staff. As an exception, Kane et al. studied nursing education and found that simulation training with mock codes was effective in terms of self-reported knowledge, skills, and personal comfort [11]. Wisniewski et al. found that nurses preferred face-to-face education, followed by online courses [12]. In needs assessment for our training approach, we found distinctly divergent learning behaviors between physicians and nurses. Physicians strongly tended to prefer self-directed learning formats and nurses preferred guided formats [13]. Most training strategies for mixed-profession teams neglect these divergent preferences, adopting a ‘one-size-fits-all’ approach.

Second, cognitive load theory [14] is highly relevant for interprofessional team training, yet often remains insufficiently emphasized. This instructional theory refers to a limited capacity of working memory of the learner, which can be overloaded by too much learning activity at the same time, inhibiting the whole learning process. It discriminates between intrinsic cognitive load – the inherent difficulty of the learning material itself – and extraneous cognitive load, which occurs due to the way the material is presented to the learner. In addition, germane cognitive load within the material aids the processing of information and transfer to long-term memory by developing schemas and frameworks. Hence, the theory postulates a simple-to-complex educational design, to foster germane cognitive load for higher learning by reducing intrinsic and extraneous cognitive load [15, 16]. Reducing intrinsic cognitive load in this context requires profession-tailored instructional approaches and individual preparation rather than mixed-group teaching only. Extraneous cognitive load must be reduced using learning methods that are proven effective. Different approaches to instructional design have been described, including blended learning approaches using e-learning resources to provide individual learning experiences [17–23]; however, effectiveness is rarely evaluated for most approaches [18]. Blended learning refers to the meaningful alignment of a conventional face-to-face teaching format enhanced by some e-learning activity, e.g. as preparation and/or wrap-up [24]. The use of virtual patients (VPs) has been shown to be effective in emergency training, especially when used in blended learning [25–28]. VP use actively involves the learner in virtual case scenarios that are enriched by multiple media and facilitate interaction and feedback [29–31]. We recently showed that the use of VPs as a preparatory tool for emergency training is effective for medical students and superior to instructional videos [32]. Moreover, VP content can easily be tailored to the specific needs of different target groups to reduce intrinsic cognitive load.

Third, in the case of an emergency, providers require clear guidelines that are valid throughout the hospital to reduce uncertainty and conflict among staff. Such appropriately designed emergency guidelines should be widely available as cognitive aids [33–35].

Using a blended approach, we designed a course on pediatric emergencies for medical and nursing staff at a tertiary academic pediatric center in Germany. The basic concept was derived from experiences in undergraduate medical training [28, 32, 36] with a blended learning approach based on in-house guidelines, profession-specific VPs as individual preparation, and simulation-based team training. The aim of this study was to evaluate participants’ perception of this approach, whether the approach improves self-assessed skills and knowledge, and what long-term effects are perceived during routine clinical practice. Improving the understanding of the potential of this educational concept might stimulate similar approaches in other clinical contexts.

## Methods

### Setting

The study was conducted at the Center for Pediatrics and Adolescent Medicine Heidelberg, a tertiary pediatric center in Germany.

### Blended training approach

We developed an interprofessional emergency training course using the established curriculum development framework by Thomas and Kern [37]. Our training course has several components (Table 1). We conducted a targeted in-house needs assessment among medical and nursing staff [13]. In-house guidelines were revised and harmonized, based on assessed needs, literature review, and in-house expert consultations. We then developed eight VPs on important topics using CAMPUS software [38], according to published design criteria [31], for their topics see Suppl. 1. Each VP was available in a ‘physician’ and a ‘nurse’ version, tailoring them to the profession-specific requirements of the case. The VPs interactively guided participants (with various questions and feedback) through an emergency encounter and were enriched with numerous pictures and video clips, as well as interactive graphics [39]. The total estimated work-up time of all preparatory VPs was 4 h. For an example description of a VP, see Table 2 in [40]. VPs were implemented in the open source learning management platform ILIAS [41]. Work-up of VPs was mandatory for participation in hands-on team training, and access was provided over four weeks prior to the practical training.

**Table 1:** Steps in the development of a blended training approach following Thomas and Kern [37]

Step 1—Problem Identification and General Needs Assessment	• Differing and/or lacking guidelines on emergencies throughout hospital departments • Need for setting up emergency training • Need for interprofessional team training
Step 2—Targeted Needs Assessment	• Identification of training content: frequent and important procedures and algorithms, as well as past issues in collaboration and communication • Identification of profession-specific learning needs • The results of this targeted needs assessment are published (13).
Step 3—Goals	• Improving and harmonizing pediatric emergency care throughout the hospital • Improving interprofessional teamwork
Step 4—Educational Strategies	• Provision of revised and harmonized guidelines throughout the hospital developed by in-house experts and literature review, making in-house guidelines available as a pocket-sized booklet • Work-up of eight VPs in profession-specific versions, enriched by various media and interactive graphics, for cognitive preparation using a blended approach • Participation in interprofessional hands-on simulation training, covering technical procedures and mock scenarios for improving collaboration and communication, tutored by interprofessional facilitator teams
Step 5—Implementation	• Distribution of in-house guideline booklets to all course participants and throughout all departments • VPs provided via web-based e-learning platform to registered course participants 4 weeks prior to team training • Eight-hour team training in interprofessional groups (two physicians and two nurses each): 4 h presenting basic procedures and an introduction to simulated scenarios; 4 h for relevant mock scenarios, including subsequent debriefing
Step 6—Concepts for Evaluating the Effectiveness of the Curriculum	• As described in this study

VP, virtual patient

**Table 2:** Overall questionnaire and results regarding training concept and components

Item		Medical staff (*N* = 55) Mean score	Nursing staff (*N* = 51) Mean score
1	The ‘pediatric emergency guidelines’ are helpful for my daily work.	4.98	4.84
2	The virtual patients were helpful for refreshing and deepening my knowledge concerning pediatric emergencies.	4.80	4.78
3	I had easy access to the virtual patients whenever I wanted.	4.80	4.71
4	Virtual patients were good preparation for the hands-on training day.	4.76	4.76
5	The scenarios of the hands-on training were realistic.	4.53	4.41
6	Tutors supported my learning success during the hands-on training.	4.84	4.88
7	The feedback I received during the hands-on training day was supportive.	4.85	4.94
8	I perceived the learning atmosphere of the hands-on training as positive.	4.89	4.84
9	By participating in the hands-on training, I improved my clinical skills in handling pediatric emergencies.	4.87	4.74
10	The hands-on training day was a worthwhile learning experience.	4.96	4.94
11	The contents of the ‘pediatric emergency guidelines,’ virtual patients, and the hands-on training day complement each other well.	4.84	4.76
12	I feel better prepared for real-life emergencies through the training.	4.78	4.51
13	Participation in the emergency training was a worthwhile learning experience overall.	4.95	4.92
14	I would recommend participating in this course to my colleagues.	4.95	4.98
15	What specific *strengths* of this training approach have you experienced?	(free text)	(free text)
16	What specific *shortcomings* of this training approach have you experienced?	(free text)	(free text)
17	What should be improved in this training approach for upcoming trainings?	(free text)	(free text)

Responses on a 5-point Likert scale from 1 ‘totally disagree’ to 5 ‘totally agree’ (items 1–14) or as free text (items 15–17)

Simulation-based team training was developed according to approved recommendations for such training [4, 9, 42]. Participants were divided into small groups of four individuals each (two physicians, two nurses) for hands-on training of 8 h. In the first half, basic measures such as pediatric basic life support including bag-valve-mask ventilation were practiced. Later, short and simple scenarios were presented, such as foreign body airway obstruction for different patient age groups. In the second half, groups rotated through four different scripted, simulated emergency scenarios and teams were subsequently debriefed by tandem tutors according to approved recommendations [42, 43]. Tutor tandems comprised a physician with experience in the pediatric intensive care unit (PICU) and a PICU nurse to ensure deliberate and interprofessional feedback. For the schedule of the training day and the scenarios used, see Suppl. 1.

All tutors participated in a 1-day workshop before the training day to become familiar with the simulation scenarios and equipment and to introduce the training concept, its goals, and the debriefing method using ‘good judgment’ [44, 45].

### Participants

Enrollment in our training was offered to all medical and nursing staff including senior staff and conducted in order of application. All enrolled participants were invited to participate during the application process. Participation in the study measures was voluntary and anonymous. Informed consent was obtained from study participants. Data were obtained either in a pseudonymized or anonymous manner, as described below, and participants could not be identified. Training capacity of the pilot run was 112 participants in total (56 medical staff, 56 nursing staff), assigned to 7 hands-on training days with four small groups each, representing the achievable study size.

### Study design

**Fig. 1:**
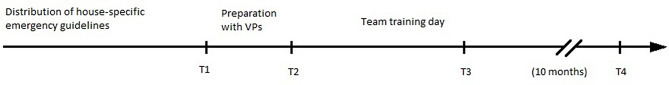
Study design and timeline. T1 to T4, study measurement points

Participants self-assessed their knowledge and skills concerning pediatric emergencies before and after preparing with the VPs (T1 and T2, respectively) and again after the hands-on training (T3) (Fig. 1). The first survey (T1) could be completed immediately before accessing the first VP, and the second (T2) was offered when completing the last VP. At T3, participants also completed a questionnaire on the training approach. Ten months after training (T4), a short follow-up survey was distributed to participants regarding the impact of the training approach and experiences in clinical work.

### Variables and measurements

#### Self-assessment

Self-assessment comprised 10 items assessing knowledge and seven items assessing clinical skills related to pediatric emergencies (Suppl. 2). The chosen items were derived from a former needs assessment among staff [13]. Items were rated on a 7-point Likert scale from 1 (‘very bad’) to 7 (‘very good’). The items were consciously kept general in order to assess these particular contents in a kind of overview. A 7-point-scale was chosen in order to provide more discrimination of changing means among a limited sample size and repetitive measurements. The electronic questionnaire was offered within the e-learning platform before (T1) and after (T2) the work-up of the preparatory VPs. After training (T3), the same questionnaire was again distributed to course participants in a paper-based format. These questionnaires were pseudonymized by self-chosen individual codes (e.g. first and second letter of place of birth, etc.) in order to identify participants who returned survey forms at all three measurement points; see description of data analysis below.

#### Questionnaire on training approach

After the training (T3), an additional anonymized questionnaire concerning the training components, the overall training, and its impact was distributed to course participants to evaluate the blended approach. This questionnaire was based on a published VP design and integration toolkit [46]. Approach-specific items were added, and non-fitting items were excluded. Fourteen items assessed training components and their integration on a 5-point Likert scale (1, ‘totally disagree’ to 5, ‘totally agree’); three free-text questions were used to query strengths, shortcomings, and possible improvements regarding the training approach (Tables 2 and 3). The questionnaire asked respondents to state their professional affiliation, but did not ask about any other personal data.

**Table 3:** Overall questionnaire and results concerning the training concept and components, repeated response categories, and selected free-text responses (items 15–17)

**15 What specific strengths of this training approach have you experienced?**	
**Medical staff**	**Nursing staff**
*Triangulation of training methods*	
‘Very good blending of emergency guidelines, virtual learning platform and implementation in the hands-on training.’ ‘Triad: computer-based training, guidelines, hands-on training; for doctors and nurses together.’ ‘Good preparation with VPs; possible to make mistakes and learn from them; joint learning for nurses and doctors; in-house guidelines can be used in training and daily work as well.’	‘Combination of preparing individually and practice under supportive supervision.’ ‘Practice with feedback by tutors, small group training, in-house guidelines, computer-based preparation for the training.’ ‘Good blending of theory and practice, concrete and close to reality.’ ‘Theoretical learning, then practice with excellent feedback.’
*Interprofessionalism*	
‘Team communication; possible to train realistically on a complete team, with all equipment; good analyses afterward.’ ‘Interprofessional exercise and teamwork, and also in stressful situations.’	‘Well experienced tutors, and tutor teams with one doctor and one nurse in each small group; preparation with VPs, guidelines for overview, and details.’
**16 What specific** ***shortcomings*** **of this training approach have you experienced?**	
**Medical staff**	**Nursing staff**
*Content*	
‘More scenarios desirable.’ ‘More kinds of emergencies desirable (e.g., 2-day hands-on training), or practicing each scenario with changing roles.’	‘More scenarios would lead to better self-confidence at the end.’
*Simulation*	
‘Manikins are always hard to assess.’ ‘Simulation of emergencies is somewhat limited’	‘Emergency manikins are still manikins.’
**17 What should be improved in this training approach for upcoming trainings?**	
**Medical staff**	**Nursing staff**
*Repetition*	
‘There should be continuous repeated training, so that freshmen in particular can participate before being on duty on their own.’ ‘Continuous repeated and refresher courses at least once a year for all medical and nursing staff.’ ‘VPs constantly available for individual refreshers.’	‘Implement the emergency training as a mandatory course for all staff, repeated every 1 or 2 years.’ ‘Mandatory course, especially for freshmen, lasting up to 2 days, for more practice.’

VP, Virtual Patient

#### Follow-up survey

After 10 months, a short anonymous survey including five items was distributed to all participants. Items covered involvement in real emergency situations after the training, as well as the impact of training on participants in these situations or beyond (Table 4). The follow-up survey was anonymous and was delivered to all participants of the training, irrespective of affiliation to medical or nursing staff.

**Table 4:** Follow-up survey items and results. *N* = 57 (both staff groups)

**1 Since participation in the emergency training, I was involved in a patient emergency** (yes/no, and free-text; 57 responses)	
Yes: 37/57 (64.9%) No: 20/57 (35.1%)	Respiratory disorder (7 mentions) Seizure (6) Anaphylaxis (3) Resuscitation (3) Sepsis (2) Cardiac syncope (1) Consciousness disorder (1) Hypovolemic shock (1)
**2 I benefited from the emergency training in a*real*emergency**,** for the following reasons** (Likert scale score for agreement and free-text categories with example answers; 36 responses)	
‘I totally agree’ or ‘I agree’ 91.7% Neutral 5.5% ‘I disagree’ or ‘I totally disagree’ 2.7%	Certainty and calm during an emergency (17 mentions) *‘I was much calmer and had more self-confidence.’* Structured educational approach (12) *‘Child with seizure*,* cyanotic and not breathing. Concrete and structured handling by physician and attending nurses; everyone knew their job.’* Improved communication (12) *‘Clear instructions to the team led to smooth handling.’* Quick response (9) *‘Sudden unexpected resuscitation situation on a peripheral ward. Extremely quick perception by the attending staff and immediate initiation of CPR and emergency call.’* Improved handling of medications and dosages (8) *‘Awareness of necessary medications and their dosages during a prolonged seizure.’* Improved clinical skills (3) *‘Certainty regarding bag-valve-mask ventilation.’* Improved adherence to algorithms (2) *‘Especially the ABCDE approach and when to call the emergency team.’*
**3 I benefited from the emergency training** ***beyond*** **emergency situations for the following reasons** (Likert scale score for agreement and free-text categories with example answers; 50 responses)	
‘I totally agree’ or ‘I agree’ 80.0% Neutral 14.0% ‘I disagree’ or ‘I totally disagree’ 6.0%	House-specific emergency guidelines (14 mentions) *‘The booklet is a good reference to look up normal values and dosages.’* Self-confidence in daily work (13) *‘I am much more confident in on-call duties when you don’t know what to expect.’* Assessment of critically ill patients (8) *‘More confident in assessing a critically ill child and deciding what to do.’* Improved communication (7) *‘I benefit a lot from closed communication*,* which saves time and leads to coordinated work.’* Improved knowledge (5) *‘Much more aware of important vital signs and immediate measures.’*
**4 If you did not benefit from the training**,** please describe what should be changed?** (free-text; no responses)	
**5 Other comments (35 responses)**	
	Repetition of training (28 mentions) *‘Yearly repetition is required to ensure replicability.’* Helpful project (10) *‘Great project*,* which must be continued.’* Self-confidence and calm (3) *‘I am much more confident and less tense in critical situations.’* VPs as refreshers for emergency preparedness (2) *‘VPs should remain available to use as a refresher.’*

Answer frequencies and categories, example free-text responses

CPR, cardiopulmonary resuscitation; ABCDE, Airway, Breathing, Circulation, Disability, Exposure

All questionnaires were pilot-tested in think-aloud sessions with volunteers from both professions to ensure the quality of the content of the questions and that the questions were properly understood, resulting in a few revisions.

### Data analysis

To create homogeneous groups for analysis, we only included self-assessments from participants who completed all three measurements. Internal consistency of the self-assessment questionnaire scores was analyzed by calculating Cronbach’s alpha for knowledge and skills scores separately for medical and nursing staff. Influences of the factor ‘group’ (medical staff, nursing staff, and both grouped together) on mean scores were calculated using factor analysis for each measurement (T1–T3) and separately for ‘knowledge’ and ‘skills’ scores. Suitability for factor analyses was confirmed using the Kaiser–Mayer–Olkin criterion (KMO) which must be 0.6 or more to be acceptable; values of 0.8 or more represent well-suited data [47]. Mean scores in each group and measurement were compared as dependent variables in a three-factor analysis of variance with the between-subjects factor ‘group’ (medical vs. nursing staff) and repeated measured factors ‘score’ and ‘measurement’ (T1–T3). Where appropriate, post-hoc tests were conducted, including Bonferroni corrections. The data were analyzed using IBM SPSS version 20 (IBM Corporation, Armonk, NY, USA). The alpha level was set at 0.05 in considering statistically significant *p* values.

We used descriptive statistics to evaluate the overall questionnaires regarding the training approach and follow-up survey. Likert scale item results are shown as mean per group, and free-text responses were evaluated using qualitative content analysis [48]. We used this inductive approach to identify key issues from general free-text responses. These categories are illustrated with representative quotes. We excluded free-text responses commenting on individual VPs, tutors, or other local circumstances.

## Results

### Participants

Fifty-six trainees from medical staff and 56 trainees from nursing staff participated in a total of 7 hands-on training days; for comparison purposes, about 120 doctors and 400 nurses were employed at that time. Self-assessments from T1 to T3 were returned by 55 medical staff trainees (98%) and 48 nursing staff trainees (85%). Questionnaires on the training approach were returned by 55 medical staff (98%) and 51 nursing staff (91%) after training (T3). From the total of 112 participants, 57 (50.9%) medical and nursing personnel returned follow-up survey forms 10 months after training.

### Self-assessments

Cronbach’s alpha values ranged from 0.839 to 0.932 (Suppl. 3), indicating good to excellent reliability [46] of the questionnaire items. Factor analyses showed relevant proportions of total variances (42.4–67.7%) and KMO test values from acceptable to very suitable for all further analyses (Suppl. 4). Factor scores and measurements were statistically significant in the three-factor analysis of variance (Suppl. 5), as well as interactions between score×group and score×measurement. A high partial Eta² of 0.755 for ‘measurement’ (Suppl. 5) indicated distinct increasing values for knowledge and skills over the three measurement time points.

**Fig. 2:**
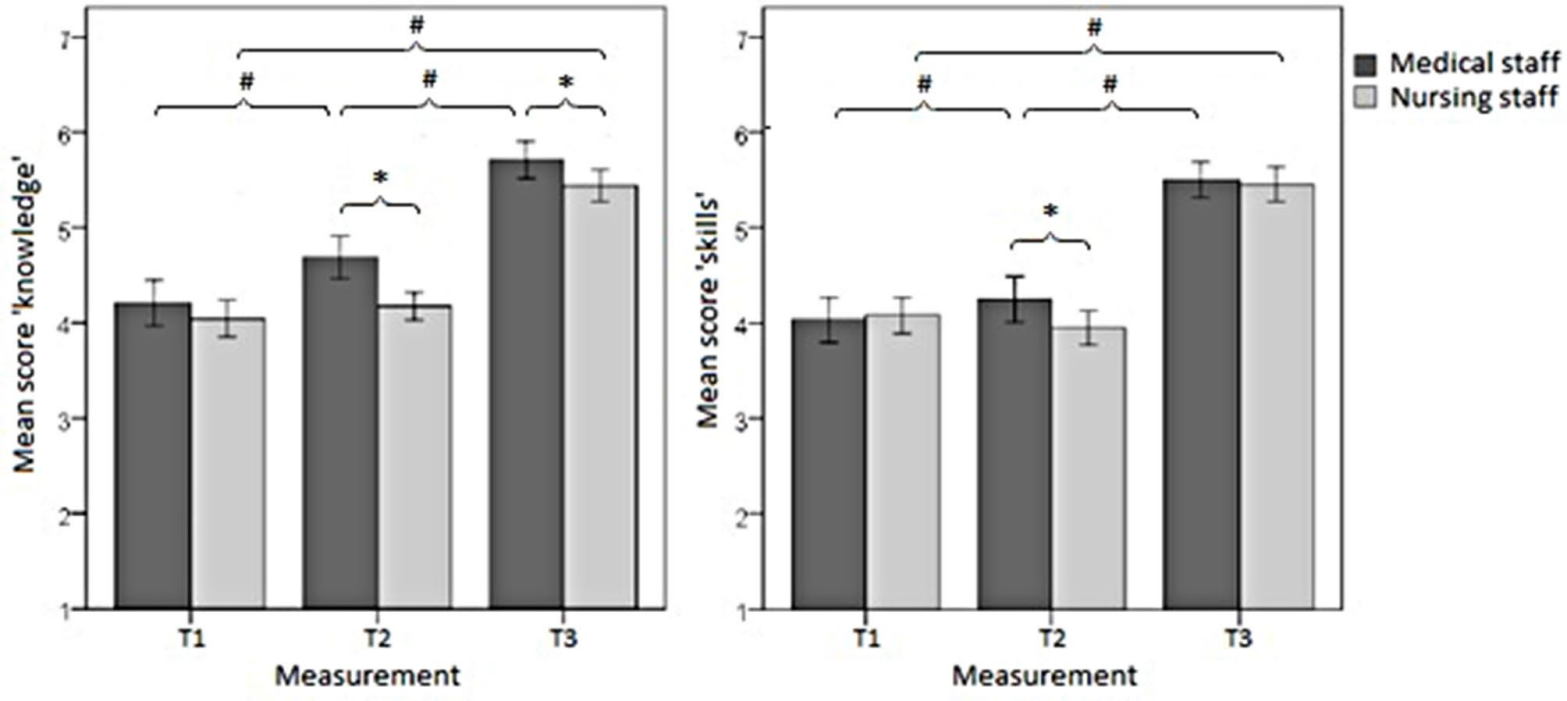
Mean scores and 95% confidence intervals for self-assessed knowledge (**a**) and skills (**b**) in the self-assessment questionnaire, completed separately by medical and nursing staff (score×group×measurement). *Significant between-group results, *p* < 0.05. ^#^Significant within-group results, *p* < 0.05

Figure 2 displays the progress from T1 to T3 regarding effects on self-assessed knowledge and skills among medical and nursing staff. Prior to preparing with VPs (T1), medical and nursing staff had comparable self-rated scores of knowledge and skills. Both groups showed statistically significant increases at T3 after training. Prior to this, after preparation with VPs at T2, only medical staff had a statistically significant increase in self-assessed knowledge and skills; nurses did not report significant improvement.

### Training approach

Table 2 shows items and results of the overall questionnaire concerning the training approach. All items received very high scores for agreement with the training approach by both medical and nursing staff. The three components of the blended training (guidelines, VPs, and team training) were perceived as helpful and participants strongly agreed that the contents complemented each other well. Participants reported feeling better prepared for real-life emergencies after the training and would recommend this course to their colleagues.

Examples of free-text responses to questionnaire items 15–17 are shown in Table 3. Strengths of the training approach were perceived in the triangulation of teaching methods and the interprofessional training and supervision. Reported shortcomings concerned the small number of different scenarios in the hands-on training, and a lack of realism of the simulation manikins. Implementation of a regular, recurrent, and mandatory course for all staff was suggested. Concerning response categories, medical and nursing staff gave similar responses to the free-text questions.

### Follow-up survey

Thirty-seven (64.9%) of the participants who returned follow-up survey forms (*N* = 57) experienced emergency situations at work during the 10 months after the training (see Table 4). Respiratory disorders and seizures were most common; a resuscitation situation was mentioned by three participants attending the same emergency encounter on a peripheral ward. Of those who experienced real emergencies, 91.7% agreed or totally agreed that they benefited from the training in these situations. Besides improved knowledge and skills concerning emergencies, improved confidence and calmness were highlighted, as well as improved communication and collaboration. Beyond emergencies situations, 80% of respondents also reported benefits from the training in daily routine practice. In particular, the developed house-specific guidelines were broadly used, and self-confidence and assessment competency regarding critically ill patients were reportedly improved. Most respondents suggested repeated training on a regular basis.

## Discussion

Overall, medical and nursing staff showed statistically significant increases in self-assessed knowledge and skills at T3 after the training. Acceptance of the training approach was high in both groups, highlighting its feasibility and the complementary nature of blended elements. In the follow-up survey, improvements were reported in patient care during real emergency encounters as well as benefits in daily clinical routines, clarifying the perceived impacts of the training on patient care.

Regarding differences by profession in terms of participants’ self-assessed increased competency over the course of this training program, we found that only medical staff had improved knowledge and skills after work-up of VPs at T2, whereas nursing staff did not. This might be because physicians are more accustomed to self-directed learning in clinical practice, as we previously observed in a needs assessment for this training at the same hospital [13]. As we measured self-reported competencies, the question arises whether this correlates with objective changes in knowledge and skills after self-guided learning, which cannot be answered in this study. Similarly, improvements of medical staff might be an overestimation. The responses given by nurses in the overall questionnaire do not support their self-reported lack of improvement in competence after the preparation, with the nurses also highlighting the VPs and the theoretical preparation. However, further studies are warranted to better understand this issue. If the reported lack of improvement in nursing staff is valid, this would call the use of VPs in nursing staff into question. However, we assume that nurses may have underestimated their competence due to the new format of self-guided learning that we introduced to our house staff. The small amount of available literature on nurses’ learning preferences (from other countries) suggests that VPs would also be an appropriate learning tool for nurses [12]. Nevertheless, compared with baseline values at T1, scores on knowledge and skills were significantly increased at T3 (after the hands-on training day) in both medical and nursing staff. At T3, physicians assessed their knowledge regarding emergencies as significantly superior to that of nurses, although their skill scores were comparable (Fig. 2).

Other researchers have evaluated emergency training effectiveness using self-assessment of knowledge and skills [11, 49]. Kane et al. found comparable results in these domains after training [11], but their approach did not include any preparatory elements like the in-house guidelines and VPs used here. Working through preparatory materials has educational advantages when individual participants are able to learn procedural knowledge in a self-directed manner and face-to-face training time can be devoted to hands-on training. This promotes repeated practice in both VP and simulated hands-on scenarios, which Issenberg et al. as well as Auerbach et al. deem necessary for long-term effectiveness [43, 49]. It also contributes to deliberate practice, which demands repeated cognitive and physical practice and provision of feedback, leading to improved performance in clinical skills [50]. The medical staff in particular perceived effective learning progress with VPs. However, self-assessed knowledge and skills can differ from external assessment, e.g., inexperience can lead to an overestimation in self-assessment, which must therefore be considered carefully [51, 52].

Preparation using VPs provides an interactive and media-enriched learning environment that fosters active learning beyond passive consumption of content, as shown in undergraduate blended learning approaches [28, 32]. VPs have been applied in undergraduate curricula in manifold ways and experiences, but are rarely studied in the area of postgraduate curricula [53], particularly interprofessional postgraduate curricula. Few reports are available on the positive impact of VPs (or any similar, case-based e-learning resources) in the context of an emergency training course, and even more rarely regarding blended learning approaches [23, 25, 54, 55].

Practice in the hands-on training day showed excellent acceptance by nursing and medical staff members alike. Participants reported feeling much better prepared for real-life emergency encounters. They emphasized that the triad of formats used – in-house guidelines, VPs, and hands-on training – complemented each other very well, along with the interprofessional small group teaching. Participants indicated a desire for more scenarios with greater realism in the simulation manikins. Among other aspects, simulation-based training allows for direct feedback, repeated practice with the possibility to increase levels of difficulty, adaptation to multiple learning strategies, individual as well as group learning, and provides a controlled and safe environment with defined outcomes and benchmarks, where errors only lead to learning and not to patient harm [43]. Manikins always have limitations in terms of depicting a real patient with all assessable senses, and usually, the more realistic a manikin, the more expensive it is to purchase and maintain. The manikins used in the training were not able, for example, to change skin color, show cyanosis or a convulsion, and could not give input to pulse oximetry or blood pressure measurement, although at least a few were high-fidelity simulators that could “speak” (via headset) and provide palpable pulse or an ECG once attached. While being more attractive to learners, high-fidelity simulators do not necessarily lead to better learning than low-fidelity simulators, and their use must be balanced with the available resources [43, 56, 57].

According to the survey responses, participants were able to transfer learning from the training to real emergencies in clinical practice, with improved interprofessional communication and collaboration, as well as enhanced confidence and preparation to deal with emergency encounters. After the training, emergency encounters were perceived as smooth and constructive in terms of team collaboration and communication, which may be interpreted as reflecting an organizational effect of the structured training approach, beyond that of the training of individuals. Improved daily work routines in the care of critically ill children throughout the hospital would be the desired goal of this work. As all of these described effects and improvements were self-reported, it should be emphasized that they naturally represent subjective perceptions only.

The strengths of this study include the blended and interprofessional design and the evaluation of perceptions both within the course and 10 months later. Limitations include the exclusive self-assessment of the generated evaluation data, which may differ from objectively measured results and can only be used as an indicator of possible effects. The evaluation instruments were not formally validated before use, although they were carefully developed, including ensuring the quality of the content of questions and the intended understanding using think-aloud sessions, and internal consistency was measured afterwards using statistical measures. Causality, e.g. for the effectiveness of the learning formats used, cannot be proven. To compare self-assessments, we did not use paired-sample testing for within-group changes, as our main focus was on between-group differences. As participation in the training as well as in the study was voluntary, this may also limit the generalizability of the findings. Transferability to other institutions and infrastructures might be restricted due to the differing professional roles and profiles of medical and nursing staff. Further studies are needed to objectively assess these effects, as well as studies to better understand the optimal blending of training methods, especially for nursing staff.

## Conclusions

We developed a novel blended learning approach to interprofessional training in pediatric emergencies that integrates self-study of hospital guidelines, VPs, and in-person simulation training, as well as debriefings. The training was perceived as effective by the participating medical and nursing staff, both immediately after the course and in subsequent real-life emergencies. This blended approach fosters deliberate practice with individualized, interactive preparation using VPs based on hospital-specific guidelines and consecutive interprofessional team training. Self-assessed competencies improved and participants reported being better prepared for real-life pediatric emergencies. Additional studies are necessary to further deepen the understanding of the optimal blending of training methods as preparation for emergency treatment.

## Electronic supplementary material

Below is the link to the electronic supplementary material.

Supplementary Material 1

Supplementary Material 2

Supplementary Material 3

Supplementary Material 4

Supplementary Material 5

## Data Availability

The datasets generated and analyzed during the current study are not publicly available but are available from the corresponding author on reasonable request.

## Abbreviations

ABCDE: Airway, Breathing, Circulation, Disability, Exposure
CPR: Cardiopulmonary resuscitation
KMO: Kaiser–Mayer–Olkin criterion
PBLS: Pediatric basic life support
PICU: Pediatric intensive care unit
SVT: Supraventricular tachycardia
T1 to T4: Study measurement points
VP: Virtual Patient
