# Supplementary material for: A novel blended and interprofessional approach to pediatric emergency training: self-assessment, perception, and perceived long-term effects
Source: BMC Med Educ. 2024 Nov 28;24:1389. doi: 10.1186/s12909-024-06381-3 (PMC11606109; doi:10.1186/s12909-024-06381-3)
Supplement: Supplementary file 1 — Supplementary Material 1 [file 12909_2024_6381_MOESM1_ESM.docx]

**Supplement 1**: Training content

**Preparatory VPs:**

VP1: Toddler with drowning accident (resuscitation incident outside the hospital)

VP2: Toddler with a sudden cough (blocked airway by foreign body aspiration outside the hospital)

VP3: Infant with emesis and diarrhea (recognition and treatment of a hypovolemic shock)

VP4: Schoolchild with sudden respiratory distress (asthmatic attack and anti-obstructive measures)

VP5: Toddler with anaphylactic reaction (recognition and treatment of anaphylaxis at all stages)

VP6: Toddler with a seizure (treatment of a status epilepticus)

VP7: Toddler with consciousness disorder (recognition and treatment of a suspected intoxication)

VP8: Schoolchild with a syncope (recognition and treatment of a cardiac arrhythmia (SVT))

**Interprofessional training day schedule**

| 8:15-8:30 | Welcoming and introduction | | | | | |
| --- | --- | --- | --- | --- | --- | --- |
|  | Room 1 | Room 2 | | Room 3 | | Room 4 |
| 8:30-  9:00 | Group A  **PBLS (1 rescuer)**  Infant | Group B  **PBLS (1 rescuer)**  Toddler | | Group C  **PBLS (1 rescuer)**  Infant | | Group D  **PBLS (1 rescuer)**  Toddler |
| 9:00-  9:30 | Group D  **PBLS (1 rescuer)**  Infant | Group A  **PBLS (1 rescuer)**  Toddler | | Group B  **PBLS (1 rescuer)**  Infant | | Group C  **PBLS (1 rescuer)**  Toddler |
| 09:30 -10:00 | Group D  **Foreign body aspiration** | Group A  **Foreign body aspiration** | | Group B  **Foreign body aspiration** | | Group C  **Foreign body aspiration** |
| 10:00-10:30 | Coffee break | | | | | |
| 10:30- 11:10 | Group C  **PBLS (2 rescuers)**  Infant | | Group D  **PBLS (2 rescuers)**  Toddler | Group A  **PBLS (2 rescuers)**  Infant | | Group B  **PBLS (2 rescuers)**  Toddler |
| 11:10-11:30 | Group B  **PBLS (2 rescuers)**  Infant | | Group C  **PBLS (2 rescuers)**  Toddler | Group D  **PBLS (2 rescuers)**  Infant | | Group A  **PBLS (2 rescuers)**  Toddler |
| 11:30-11:45 | Group activity | | | | | |
| 11:45-12:45 | Lunch break | | | | | |
| 12:45-13:00 | Scenario introduction | | | | | |
| 13:00-13:50 | Group A  **Scenario 1** | | Group B  **Scenario 2** | | Group C  **Scenario 3** | Group D  **Scenario 4** |
| 13:50 -14:40 | Group D  **Scenario 1** | | Group A  **Scenario 2** | | Group B  **Scenario 3** | Group C  **Scenario 4** |
| 14:40-15:00 | Coffee break | | | | | |
| 15:00-15:45 | Group C  **Scenario 1** | | Group D  **Scenario 2** | | Group A  **Scenario 3** | Group B  **Scenario 4** |
| 15:45-16:30 | Group B  **Scenario 1** | | Group C  **Scenario 2** | | Group D  **Scenario 3** | Group A  **Scenario 4** |
| 16:30-17:00 | Feedback round and discharge | | | | | |

*Used scenarios:*

Scenario 1: Dehydrated infant on ward – (hypovolemic) shock recognition and treatment, patient admitted due to severe febrile infection

Scenario 2: Toddler with epileptic seizure in the outpatient clinic – first episode of a prior unknow epileptic disorder

Scenario 3: Infant with resuscitation incident on ward – septic shock led to cardiac failure

Scenario 4: Toddler with asthmatic attack in the outpatient clinic – know asthma patient with increasing respiratory distress
