# Supplementary material for: A novel blended and interprofessional approach to pediatric emergency training: self-assessment, perception, and perceived long-term effects
Source: BMC Med Educ. 2024 Nov 28;24:1389. doi: 10.1186/s12909-024-06381-3 (PMC11606109; doi:10.1186/s12909-024-06381-3)
Supplement: Supplementary file 2 — Supplementary Material 2 [file 12909_2024_6381_MOESM2_ESM.docx]

**Supplement 2**: Items on the self-assessment questionnaire

| **Knowledge** | **Skills** |
| --- | --- |
| I rate my present knowledge on … as | I rate my present clinical skills on … as |
| - the ABCDE scheme - pediatric basic life support - management of anaphylaxis - management of foreign body airway obstruction - prolonged seizure - nondistinctive consciousness disorder - lack of volume shock - respiratory distress in asthmatic attack - cardiocirculative disorder - communication principles in emergency situations | - clinical application of the ABCDE scheme - pediatric basic life support - bag-valve-mask ventilation of a child - providing appropriate measures in foreign body airway obstruction - leading the team in an emergency encounter (medical staff), preparation and drawing up of emergency medications (nursing staff), respectively - effective communication within a team during an emergency - pointing out treatment errors to the team |

Responses given on a 7-point scale from 1, ‘very bad’ to 7, ‘very good.’
