# Supplementary material for: A novel blended and interprofessional approach to pediatric emergency training: self-assessment, perception, and perceived long-term effects
Source: BMC Med Educ. 2024 Nov 28;24:1389. doi: 10.1186/s12909-024-06381-3 (PMC11606109; doi:10.1186/s12909-024-06381-3)
Supplement: Supplementary file 3 — Supplementary Material 3 [file 12909_2024_6381_MOESM3_ESM.docx]

**Supplement 3**: Cronbach’s alpha values for self-assessment questionnaire results

|  | T1 | | T2 | | T3 | |
| --- | --- | --- | --- | --- | --- | --- |
|  | Knowledge | Skills | Knowledge | Skills | Knowledge | Skills |
| Medical staff (N=55) | 0.915 | 0.859 | 0.932 | 0.917 | 0.928 | 0.890 |
| Nursing staff (N=48) | 0.858 | 0.840 | 0.839 | 0.844 | 0.890 | 0.911 |
| Total (N=103) | 0.896 | 0.842 | 0.916 | 0.891 | 0.916 | 0.896 |
