# Supplementary material for: A novel blended and interprofessional approach to pediatric emergency training: self-assessment, perception, and perceived long-term effects
Source: BMC Med Educ. 2024 Nov 28;24:1389. doi: 10.1186/s12909-024-06381-3 (PMC11606109; doi:10.1186/s12909-024-06381-3)
Supplement: Supplementary file 4 — Supplementary Material 4 [file 12909_2024_6381_MOESM4_ESM.docx]

**Supplement 4**: Factor (group)-dependent proportions of variances in scores on self-assessment questionnaire

|  | T1 | | T2 | | T3 | |
| --- | --- | --- | --- | --- | --- | --- |
|  | Knowledge | Skills | Knowledge | Skills | Knowledge | Skills |
| Medical staff (N=55) | 57.4%  (0.868) | 55.9%  (0.787) | 63.0%  (0.876) | 67.5%  (0.837) | 62.0%  (0.890) | 61.8%  (0.789) |
| Nursing staff (N=48) | 45.2%  (0.798) | 53.0%  (0.814) | 42.4%  (0.784) | 53.5%  (0.726) | 51.7%  (0.843) | 66.3%  (0.838) |
| Total (N=103) | 52.5%  (0.887) | 52.8%  (0.835) | 58.0%  (0.902) | 67.6%  (0.834) | 57.7%  (0.897) | 62.5%  (0.818) |

Kaiser–Meyer–Olkin test values in parentheses.
