# Supplementary material for: A novel blended and interprofessional approach to pediatric emergency training: self-assessment, perception, and perceived long-term effects
Source: BMC Med Educ. 2024 Nov 28;24:1389. doi: 10.1186/s12909-024-06381-3 (PMC11606109; doi:10.1186/s12909-024-06381-3)
Supplement: Supplementary file 5 — Supplementary Material 5 [file 12909_2024_6381_MOESM5_ESM.docx]

**Supplement 5**: Three-factor analysis of variance, dependent variable (mean score)

| **Source** | **Type III sum of squares** | **df** | **Mean squared** | **F** | **Sig.** | **Partial eta squared** |
| --- | --- | --- | --- | --- | --- | --- |
| Repeated measures effects | | | | | | |
| Score | 4.122 | 1 | 4.122 | 20.318 | .000 | .163 |
| Score×Group | 1.824 | 1 | 1.824 | 8.989 | .003 | .080 |
| Error (Score×Group) | 21.100 | 104 | .203 |  |  |  |
| Measurement | 260.933 | 2 | 130.467 | 321.211 | .000 | .755 |
| Measurement×Group | 3.412 | 2 | 1.706 | 4.201 | .016 | .039 |
| Error (Measurement×Group) | 84.484 | 208 | .406 |  |  |  |
| Score×Measurement | 2.276 | 2 | 1.138 | 12.441 | .000 | .107 |
| Score×Measurement×Group | .002 | 2 | .001 | .009 | .991 | .000 |
| Error (Score×Measurement×Group) | 19.027 | 208 | .091 |  |  |  |
| Between-subject effects | | | | | | |
| Group | 6.825 | 1 | 6.825 | 3.389 | .068 | .032 |
| Error | 209.495 | 104 | 2.014 |  |  |  |
